# Finger Citron Extract Ameliorates Glycolipid Metabolism and Inflammation by Regulating GLP-1 Secretion via TGR5 Receptors in Obese Rats

**DOI:** 10.1155/2021/6623379

**Published:** 2021-03-27

**Authors:** Yujiao Yang, Aofei Tian, Zhen Wu, Yao Wei, Xuguang Hu, Jiao Guo

**Affiliations:** ^1^Guangdong Metabolic Disease Research Center of Integrated Chinese and Western Medicine, Guangdong Pharmaceutical University, Guangzhou, China; ^2^Department of Pharmacology of Chinese Materia Medica, School of Traditional Chinese Medicine, Guangdong Pharmaceutical University, No. 280, East Ring Road, Guangzhou University Town, Guangdong 510006, China; ^3^Guangdong Metabolic Diseases Research Center of Integrated Chinese and Western Medicine, Key Laboratory of Glucolipid Metabolic Disorder, Ministry of Education of China, Institute of Chinese Medicine, Guangdong Pharmaceutical University, Guangdong TCM Key Laboratory for Metabolic Diseases, Guangzhou 510006, China

## Abstract

Finger citron (FC) is one of many traditional Chinese herbs that have been used to treat obesity. The aim of this study was to elucidate the pharmacological effects and mechanisms of FC on obese rats. Rats were fed with a high-fat diet as a model of obesity and treated with FC at three different dosages for 6 weeks. Pathology in liver tissue was observed. Glucose levels, lipids levels, and inflammatory indicators in serum were evaluated by enzyme‐linked immunosorbent assay. Furthermore, the expression of *G* protein-coupled receptor 5 (TGR5) pathway genes in rat colon tissue was detected by reverse transcription-polymerase chain reaction analysis (RT-PCR). Our result revealed that FC alleviates obesity by reducing body weight (BW) and waist circumference, managing inflammation and improving glycolipid metabolism, liver function, and liver lipid peroxidation in vivo. In addition, the mechanism of FC on obesity is possibly the stimulation of glucagon-like peptide-1 (GLP-1) secretion by activating the TGR5 pathway in intestinal endocrine cells. Our studies highlight the obesity reduction effects of FC and one of the mechanisms may be the activation of the TGR5 pathway in intestinal endocrine cells.

## 1. Introduction

Obesity is a complex metabolic condition with attendant disorders that poses a significant risk of morbidity and mortality on a global scale [1]. It is associated with multiple comorbidities involving glycolipid metabolism, insulin resistance, and dyslipidemia, as well as chronic inflammation, oxidative stress, and liver diseases [2, 3]. Patients commonly prefer pharmacotherapy or surgical approaches rather than a healthy diet and exercise [4]. However, pharmaceutical drugs such as orlistat often cause gastrointestinal adverse effects and even liver and kidney injury. Bariatric surgeries are expensive and have uncertain long-term and certain negative effects [5]. Glucagon-like peptide-1 (GLP-1), which is released from gut enteroendocrine cells, not only augments insulin and inhibits glucagon secretion to control meal-related glycemic excursions but also decreases gastric emptying and food intake to limit weight gain [6]. GLP-1 receptor agonists have recently been confirmed to promote glucose control and body weight loss [7]. Consequently, GLP-1 and its analogues have been increasingly recognized as a hot pot in the medical field for obesity and type 2 diabetes [8].

Fatty infiltration of the liver increases liver oxidative stress and inflammation, which could affect liver function [9]. Fatty liver is associated with the expression of proinflammatory cytokines such as tumor necrosis factor-*α* (TNF-*α*) and interleukin-6 (IL-6) and with elevated levels of alanine transaminase (ALT) and aspartate transaminase (AST) [10]. Oxidative stress can undermine normal cells by activating nuclear factor-kappa B inhibitor kinase (NF-*κ*B) and other signaling pathways, leading to the development of insulin resistance or inflammatory response [11, 12]. Therefore, oxidative stress may be one of the causes of obesity. Low-grade inflammation caused by obesity is a long-term, low-level systemic inflammatory response [13]. It was demonstrated that chronic inflammation persisted throughout the pathogenesis of obesity and insulin resistance [14]. Oxidative stress can be eliminated by endogenous anti-oxidative enzymes such as superoxide dismutase (SOD) [15].

G protein-coupled receptor 5 (TGR5) on enteroendocrine L cells promotes the secretion of, among others, glucagon-like peptide-1 (GLP-1) [16, 17], an incretin hormone that can regulate glucose by exerting insulinotropic action [18]. When activated by bile acid or TGR5 agonists, TGR5 induces the release of intracellular second messengers like cyclic adenosine monophosphate (cAMP), which in turn regulates NF-*κ*B and protein kinase A activities. Inhibition of nuclear factor NF-*κ*B limits the secretion of proinflammatory cytokines such as TNF-*α* and IL-6 [19, 20].

Fingered citron, *Citrus medica* L. var. *sarcodactylis* Swingle, is the dried fruit of the bergamot of the family Rutaceae. It has been used in treating various chronic diseases and as a food additive or nutritional supplement, given its abundance in phenolic compounds that exhibit high antioxidant activity [21, 22]. Mollace et al. [23] found that its extract can reduce total cholesterol and triglycerides in rats under diet-induced hyperlipidemia. Peng et al. [24] found that FC could lower blood sugar and improve glucose tolerance to treat type 2 diabetic rats. Kabra et al. [25] found that ethanolic extract of FC peels possessed potent antioxidant and antidiabetic properties. Impellizzeri et al. [26] found that FC juice extract could improve inflammatory bowel disease. Hesperidin, an important chemical compound found in citrus fruits, has shown strong anti-inflammatory activity [27]. A preliminary study we conducted showed that hesperidin remarkably decreased glucose content and increased glucose uptake in insulin-resistant human hepatocellular carcinoma (HepG2) cells. Furthermore, we found that hesperidin ameliorated insulin resistance by regulating the insulin receptor substrate 1-glucose transporter 2 pathway via toll-like receptor 4 in HepG2 cells [28].

Although there are many studies on the composition of finger citron these years, the research of its pharmacological effects and mechanisms on regulating metabolism disorders remains scarce. FC, a natural herb with multiple uses, is promised to have important implications for the treatment of glycolipid metabolism. Thus, the main aim of this study was to elucidate the pharmacological effects and mechanisms of FC on obese rats.

## 2. Materials and Methods

### 2.1. Chemicals and Reagents

Dried FC was purchased from Beijing Tongrentang Guangzhou Pharmaceutical Chain Co., Ltd. (Beijing, China) and was certified by Professor Li Shuyuan of the Department of Traditional Chinese Medicine Identification, College of Traditional Chinese Medicine, Guangdong Pharmaceutical University, in accordance with the requirements of the Pharmacopoeia of the People's Republic of China, 2015 Edition (Chinese Pharmacopeia). FC extract was centrifuged at 6000 rpm/min for 15 min to remove any impurities and subsequently transformed into a dry powder by spray-drying. Small aliquots of FC were stored at 20°C. The resulting drug was thawed and dissolved in non-pyrogenic saline just prior to use. High-performance liquid chromatography revealed 32 known components such as naringin, hesperidin, 6,7-dimethoxycoumarin, 5,7-dimethoxycoumarin, and bergapten [29].

Glucose kits, TC, TG, high-density lipoprotein cholesterol (HDL-C), low-density lipoprotein cholesterol (LDL-C), SOD, malondialdehyde (MDA), ALT, and AST were obtained from Nanjing Jiancheng Bioengineering Institute (China). D-LAC enzyme-linked immunosorbent assay (ELISA) kits, total bile acid, GLP-1, LPS (lot number MM-0647R1), TNF-*α* (lot number MM-0517R1), IL-6 (lot number MM-0521R1), and interleukin 1 beta (IL-1*β*; lot number MM-0626R1) were purchased from Biotechnology Co., Ltd., Shanghai Enzyme Research (China). Bicinchoninic acid (BCA) Protein Quantification Test Kits were purchased from Beyotime Institute of Biotechnology (Shanghai, China). TGR5 and GLP-1 antibodies were purchased from Abcam, Inc. (UK). *β*-Actin and horseradish peroxidase- (HRP-) labeled goat anti-rat antibodies were purchased from Wuhan Servicebio Technology Co., Ltd. (China). All other solvents reagents were of analytical grade.

### 2.2. Animals and Diets

60 healthy 5-week-old male Sprague-Dawley rats weighting about 140–160 g were purchased from the Experimental Animal Center of Southern Medical University, experimental animal certificate number: SCXK (Yue) 2016–0041. All experimental procedures were carried out following internationally accepted principles for laboratory animal use and care and were approved by Ethical Committee for Animal Experimentation of the Guangdong Pharmaceutical University (Guangdong, China). Normal diet contained corn, soya, and vitamins. The high-fat purified diet was comprised of 60 kcal % fat and purchased from Dyes, America (No. 112252). Each cage was covered with poplar padding and housed five rats. The cages were placed in an SPF-grade, sterile laminar flow, and climate-controlled environment (22°C ± 2°C at 50% relative humidity) with a 12 h light, 12 h dark cycle. The rats with available water and food were acclimatized to the standard environmental conditions for 7 days prior to the experiment. All experiments involving animals were approved by the Ethical Committee for Animal Experimentation of the Guangdong Pharmaceutical University (Guangdong, China).

The obese model was considered accomplished when the average weight of the rats fed with high-fat diet surpassed 20% of that of the normal diet. Then, the obese rats were randomly divided into five groups of 10 animals each: high‐fat diet group (HFD), orlistat group treated with orlistat (75 mg/kg/day) (orlistat) [30], low dose of FC (FC-L), medium dose of FC (FC-M), and high dose of FC (FC-H). ND and HFD received a gavage of 1 ml water daily. Orlistat group received a gavage of 75 mg/kg/day of orlistat. FC-L received a gavage of 0.55 g/kg/day of FC. FC-M received a gavage of 1.1 g/kg/day of FC. FC-H received a gavage of 2.2 g/kg/day of FC. The gavage lasted for 6 weeks. Weekly body weight was recorded using an electronic scale and waist circumstance was measured using a standard measuring tape around the anterior abdomen in centimetre [31]. The measurements were carried out under light anaesthesia using amobarbital. Each group had free access to water during the experimental period. The dosage conversion method of FC was based on a standard human adult dose to mouse body surface area in the Chinese Pharmacopoeia 2015 Edition. The conditions of normal group rats (ND) always remained unchanged.

### 2.3. Preparation of Serum

After 6 weeks of gavage, rats were weighed and then anaesthetized with 80 mg/kg amobarbital via intraperitoneal injection. Then, a thoracotomy was performed and blood was drawn via cardiac puncture until death. Before taking blood, the rats were fasted for 24 h, and litter was removed to eliminate the effect of feed debris on fasting blood parameters. After anesthetizing the rats with ether, the tails were cut from the vein on both sides and a clean Eppendorf (EP) tube was used to hold the blood. The tubes were kept at 24°C for 1 h, centrifuged at 3500 rpm/min for 10 min at 4°C to obtain serum, and subsequently stored at −80°C. The centrifuged blood samples were not thawed after freezing during storage. The levels of TNF-*α*, IL-6, TC, TG, FBG, SOD, and MDA in the supernatant were measured using ELISA kits according to the manufacturer's instructions.

### 2.4. Liver Hematoxylin and Eosin (HE) and Oil Red O Staining

Liver samples were taken and immediately fixed in 10% formalin, embedded in paraffin, and stained with hematoxylin and eosin (HE) and Oil Red O, then embedded in paraffin, and lastly cut into 5 *μ*m thick sections using a Leica RM2235 microtome (Leica, Heidelberg, Germany). The tissues were stained with HE and observed under a microscope at 200× or 400×.

### 2.5. Enzyme-Linked Immunosorbent Assay (ELISA)

Serum measurements were performed before and after vitamin *D* supplementation to determine initial and terminal vitamin *D* levels using individual ELISA kits (Eastbiopharm, Zhejiang, China) according to the manufacturer's instructions. Tissue samples were homogenized in phosphate-buffered saline and centrifuged at 10000*g* at 4°C for 20 min. Clear supernatants were collected, and the total protein concentration was measured using a protein assay kit (Pars Azmun, Tehran, Karaj) (Hangzhou Eastbiopharm, Zhejiang, China). D-LAC, Total bile acid, GLP-1, LPS TNF-*α*, IL-6, and IL-1*β* concentration in the supernatants were determined using ELISA purchased from Biotechnology Co., Ltd., Shanghai Enzyme Research (China).

### 2.6. Reverse Transcription-Polymerase Chain Reaction (RT-PCR) Analysis

Total RNA was isolated from liver or adipose tissue samples using TRIzol (Invitrogen, Inc., Carlsbad, CA, USA). Single-stranded cDNA was generated from 1 *μ*g of total RNA using TaKaRa PrimeScript™ RT reagent kit. The cDNA products were amplified by real-time RT-PCR using the TaKaRa SYBR Premix Ex Taq™ kit and the Bio-Rad IQ5 real-time PCR system. The Bio-Rad real-time PCR analysis software (Applied Biosystems, Carlsbad, CA, USA) was used for data collection and analysis. The primer sequences (Table 1) used for PCR were synthesized by Sangon Biotech Co. Ltd. (Shanghai, China), and *β*-actin was used as the internal control (housekeeping gene).

### 2.7. Statistical Analysis

Statistical analyses were performed using SPSS 24.0 software. All results were presented as mean ± standard deviation (SD) and plotted using GraphPad Prism 7.0 software. Analysis of variance (one-way ANOVA) was performed, followed by the Student–Newman–Keuls test for significance. The level of significant differences between means was set at *P* < 0.05.

## 3. Results

### 3.1. FC Reduced Body Weight, Waist Circumference, and Lipid Deposition in Obese Rats

**Figure 1:**
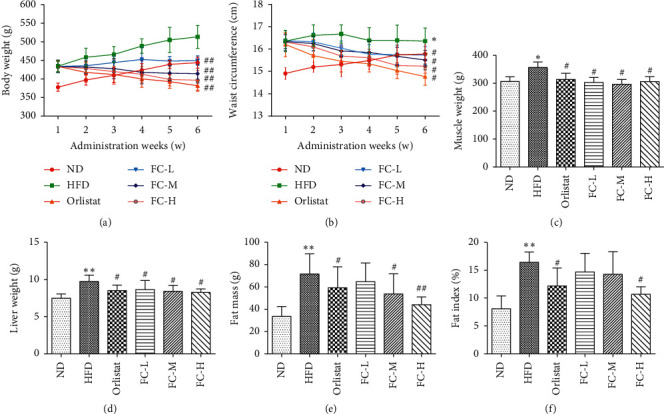
FC alleviated body weight, waist circumference, and lipid deposition of obese rats. Data are presented as the means ± SD (*N* = 8).  ^*∗*^*P* < 0.05 and  ^*∗∗*^*P* < 0.01 versus the normal group (ND).  ^#^*P* < 0.05 and  ^##^*P* < 0.01 versus the high-fat diet group (HFD).

Rats were monitored for body weight and waist circumstance per week. Body weight, waist circumference, muscle mass, fat mass, liver mass, and fat index of HFD rats remarkably increased compared with ND rats (*P* < 0.05, Figures 1(a)–1(f)). Variety of body weight and waist circumference during gavage, respectively, reflected effects of FC on obesity (Tables 2 and 3). However, the HFD group did not reach a significant reduction in waist circumstance at the final intervention week compared with ND (Figure 1(b)). We speculated that the waist circumference of HFD would remain unchanged after reaching a certain measurement level, whereas that in the normal group continued to increase. The fat indexes in the three FC groups showed different degrees of decrease (Figure 1(f)). These results suggest that FC reduced body weight, waist circumference, and lipid deposition in obese rats.

### 3.2. FC Lowered Blood Lipid and Glucose Levels in Obese Rats

**Figure 2:**
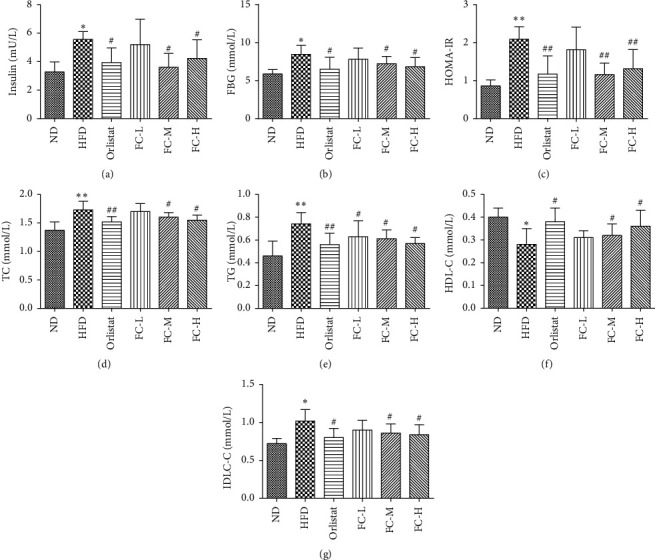
Fingered citron abolished glycolipid metabolism in obese rats. Data are presented as the means ± SD (*N* = 8).  ^*∗*^*P* < 0.05 and  ^*∗∗*^*P* < 0.01 versus the normal group (ND).  ^#^*P* < 0.05 and  ^##^*P* < 0.01 versus the high-fat diet group (HFD).

As is shown in Figure 2, in contrast to those in the ND group, the TC, TG, LDL-C, FBG, and insulin levels and homeostatic model assessment of insulin resistance (HOMA-IR) in the serum of HFD rats were significantly increased (*P* < 0.05) with the HDL-C levels decreased (*P* < 0.05). This indicates that a long-term, high-fat diet contributed to glycolipid metabolism. In the HFD group, the HDL-C levels were significantly increased (*P* < 0.05), whereas those in the orlistat, FC-M, and FC-H groups were significantly lower (*P* < 0.05). All indexes of the FC-L group decreased compared with HFD, showing no significant differences except in TG levels. The results suggest that FC could reduce glycolipid metabolism in obese rats.

### 3.3. FC Reversed Inflammation in Obese Rats

**Figure 3:**
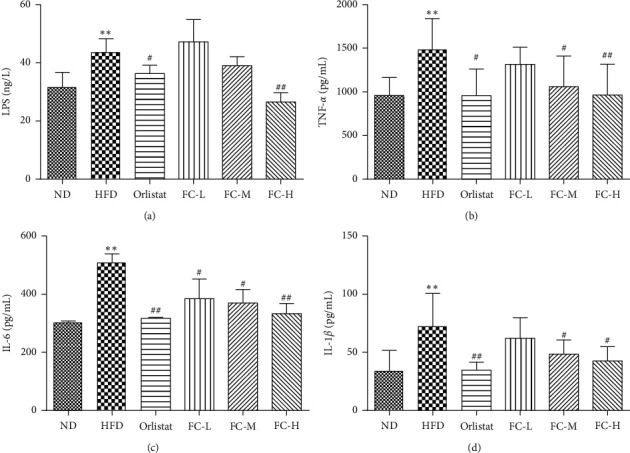
Effects of fingered citron on plasma endotoxin and intestinal inflammatory factors in rats. Data are presented as the means ± SD (*N* = 8).  ^*∗*^*P* < 0.05 and  ^*∗∗*^*P* < 0.01 versus the normal group (ND).  ^#^*P* < 0.05 and  ^##^*P* < 0.01 versus the high-fat diet group (HFD).

As shown in Figure 3, compared with ND, the lipopolysaccharide (LPS), TNF-*α*, IL-6, and IL-1*β* levels in the plasma of HFD rats were significantly increased (*P* < 0.05). Compared with those of HFD, LPS levels of the orlistat and FC-H groups were significantly lower (*P* < 0.01). TNF-*α*, IL-6, and IL-1*β* levels in colon tissue of the orlistat, FC-M, and FC-H groups were significantly decreased (*P* < 0.05). These results suggest that FC can attenuate low-grade inflammation in obese rats.

### 3.4. FC Improved Liver Lipid Deposition, Peroxidation, and Overall Function in Obese Rats

**Figure 4:**
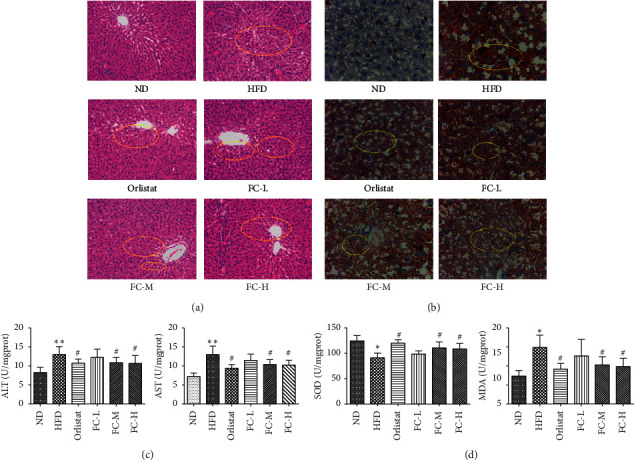
FC improves liver lipid deposition, liver peroxidation, and liver function in obese rats. Representative photographs of liver histopathological changes in rats by HE staining (×200) and oil red O staining (×400). Data are presented as the means ± SD (*N* = 8).  ^*∗*^*P* < 0.05 and  ^*∗∗*^*P* < 0.01 versus the normal group (ND).  ^#^*P* < 0.05 and  ^##^*P* < 0.01 versus the high-fat diet group (HFD).

As shown in Figure 4, the liver lobules of rats in the ND group were structurally intact with a clear outline, and the hepatic sinus was elongated and regularly shaped. The hepatic cord was arranged radially in the center of the central vein, and the hepatocyte cytoplasm was uniform (Figure 4). In contrast, the hepatic lobular structure of rats on HFD is not clear. The hepatocyte volume is increased, and circular vesicles (lipid droplets) of different sizes can be seen in the cytoplasm of the cells. Furthermore, nuclei were pushed to the periphery with focal inflammatory cell infiltration. The hepatic cord arrangement was disorderly because of severe hepatocyte degeneration, which is characteristic of fatty liver (indicated by the yellow circle). The orlistat, FC-L, FC-M, and FC-H groups showed less inflammatory cell infiltration than the HFD. The hepatic cords arrangement was neater, and lipid droplets were fewer. Some cells still showed water-like degeneration but most had varying degrees of recovery (shown by yellow circles).

Compared with those in ND, SOD activity in the HFD liver samples significantly decreased (*P* < 0.05), whereas the MDA, ALT, and AST levels significantly increased (*P* < 0.05), suggesting that HFD promotes abnormal liver function and significant lipid peroxidation. In contrast, the MDA, ALT, and AST levels in the orlistat, FC-M, and FC-H groups significantly decreased (*P* < 0.05), and SOD activity significantly increased (*P* < 0.05). Meanwhile, levels of the abovementioned parameters in the FC-L group decreased to a certain extent without significant difference (*P* > 0.05). The results suggest that FC has certain anti-oxidative and anti-inflammatory properties.

### 3.5. Effects of FC on Expression of TGR5, cAMP, and NF-*κ*B Genes in Colon Tissue

**Figure 5:**
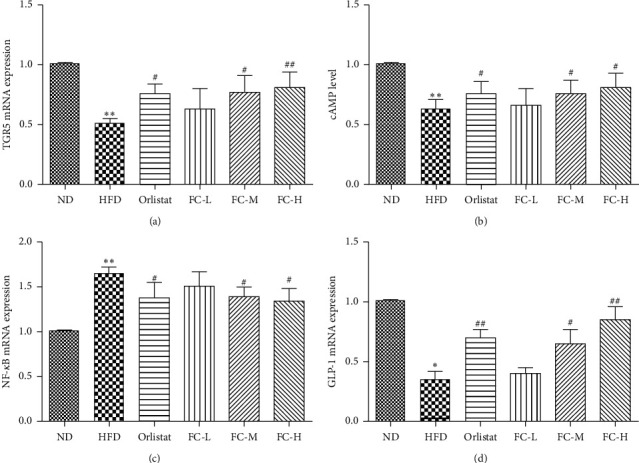
Effect of fingered citron on colon tissue expression of TGR5, cAMP, and NF-*κ*B in rat. Data are presented as the means ± SD (*N* = 8).  ^*∗*^*P* < 0.05 and  ^*∗∗*^*P* < 0.01 versus the normal group (ND).  ^#^*P* < 0.05 and  ^##^*P* < 0.01 versus the high-fat diet group (HFD).

As shown in Figure 5, the genetic expression of TGR5 and cAMP in colon tissue of HFD rats significantly decreased (*P* < 0.01), whereas the genetic expression of NF-*κ*B significantly increased (*P* < 0.05). Compared with HFD, the expression of TGR5 and cAMP genes in colon tissue of the orlistat, FC-M, and FC-H groups significantly increased (*P* < 0.05), whereas the genetic expression of NF-*κ*B significantly decreased (*P* < 0.05). These findings indicate that FC can upregulate the genetic expression of TGR5 and cAMP and inhibit the genetic expression of NF-*κ*B in colon tissue.

## 4. Discussion

The study demonstrated that FC may have effective anti-obesity mechanisms in vivo. The effects of FC on obesity may include reducing body weight, waist circumference, adipose deposition, and inflammation, alleviating liver dysfunction and oxidative stress, and activating TGR5 in obese rats.

FC has been long used as traditional Chinese medicinal material and functional vegetables. FC fruits belonging to the family Rutaceae, commonly known as “five finger orange” in commercial vegetable markets, are an important functional food source in a specific diet [32]. Researchers [24] carried out simultaneously an acute oral safety test with rats to investigate whether FC fruits can be effective and safe. The result showed that 2000 mg/kg per os of FC fruits was totally nontoxic in nature despite its ripening stage.

Obesity is one of the global public health challenges and the most prevalent chronic metabolic disorder. Reduced insulin sensitivity and impaired glucolipid uptake in obese patients induce hyperglycemia and hyperlipidemia [33]. Miceli et al. found that FC can significantly reduce the levels of TC, TG, and LDL-C in the serum of hyperlipidemic rats and increase HDL-C [34]. Zhang et al. showed that hesperidin could increase the glucose consumption of HepG2 cells by enhancing the phosphorylation of adenosine monophosphate-activated protein kinase, which plays an important role in regulating the blood sugar levels [35]. The present study demonstrated that FC increased HDL-C and decreased FBG, insulin, HOMA-IA, TC, TG, and LDL-C in obese rats, not to mention its reduction of fat index levels. Thus, FC could improve glycolipid metabolism and insulin resistance.

Inflammatory pathways in obesity prompt oxidative stress. The increased oxidative stress in accumulated fat promotes the pathogenesis of obesity-associated metabolic syndrome [36]. TNF-*α*, IL-6, and IL-1*β* are the most important cytokines involved in the pathogenesis of colitis [37]. Impellizzeri et al. [26] confirmed that the expression of TNF-a and IL-1*β* reduced in the colon tissues of inflammatory bowel rats. Meloni et al. [38] found that flavonoids in FC can inhibit TNF-*α* and IL-8 stimulated by LPS in monocytes. Kim et al. [39] found that essential oils and their constituents from FC had anti-inflammatory properties, inhibiting the activation of the NF-*κ*B pathway. The present study showed that FC could regulate LPS, TNF-*α*, IL-6, and IL-1*β* levels in serum of obese rats.

The liver is the most important metabolic organ in the human body. It also plays an important role in oxidative stress, storage of liver glycogen, digestion, and immunity, among others. Liver function also directly affects the body's lipid metabolism level [40]. In our study, FC could significantly inhibit TC, TG, ALT, AST, and MDA while promoting SOD. Furthermore, it protects liver function by resisting liver lipid peroxidation in obese rats.

GLP-1 analogues are currently used for type 2 diabetes treatment. A more in-depth study of the mechanisms of endogenous GLP-1 release may facilitate the development of selective GLP-1 agonists [41]. TGR5 is a G protein-coupled receptor on intestinal endocrine cells. Bile acids can specifically bind to the TGR5 receptor [42], which can activate cAMP signaling. In turn, cAMP inhibits the activation of NF-*κ*B, as well as the inflammatory response. A number of studies have established that NF-*κ*B plays a central role in the regulation of various genes responsible for the generation of mediators or proteins in ulcerative colitis [43]. Trombetta et al. found that FC peel extracts significantly decreased NF-kB levels in human umbilical vein endothelial cells [44]. TGR5 can also stimulate the secretion of GLP-1 in intestinal endocrine cells, thereby stimulating islet *β* cells to secrete insulin and inhibit the secretion of glucagon, thereby lowering blood sugar and improving glucose metabolism [45]. TGR5 inhibition could reduce cAMP and GLP-1 secretion in NCI–H716 cells [46]. TGR5 also partly inhibits NF-*κ*B signaling in gastric inflammation [47]. The experimental results in this section are consistent with these studies. As the results of this study show, the rats in HFD showed dysfunctional glucose and lipid metabolism, insulin resistance, and low-grade inflammation. After FC administration, intestinal mucosal permeability was decreased.

## 5. Conclusion

Our study indicated that fingered citron could significantly reduce fat of obese rats and improve the disorder of glycolipid metabolism, inflammation, liver dysfunction, and oxidative stress in obese rats. Moreover, we validated the possible mechanisms by which FC effectively ameliorated obesity might be activating TGR5 regulation so as to ameliorate the glycolipid metabolism disorder as well as inflammation. Therefore, the study suggests a beneficial effect of FC in improving obesity and its metabolic syndrome components. Since the prevalence of obesity becomes common in the population worldwide, FC supplementation may serve as a complementary dietary strategy of GLP-1 receptor agonists to manage obesity.

## Figures and Tables

**Table 1:** The primer sequences used for PCR measurements.

Name	Sequences
TGR5	Forward: 5′-TCATCGTCATCGCCAACCT-3′
	Reverse: 5′-CCCAGCTAGTAGTAGGCTTAGAAAG-3′
NF-*κ*B	Forward: 5′-GACACGACAGAATCCTCAGCATCC-3′
	Reverse: 5′-GCCACCAGCAGCAGCAGAC-3′
GLP-1	Forward: 5′-TGCGGATCCCACGGTGAAGGTACTTTCACTTCT GA-3′
	Reverse: 5′-ATAGCGGCCGCTTAACGACCCTTAACCAACCAAGC-3′

**Table 2:** Effects of FC on body weight each week during gavage.

	1th	2nd	3rd	4th	5th	6th
ND	377.78 ± 11.13	397.38 ± 13.72	409.85 ± 19.86	423.67 ± 17.33	439.22 ± 10.69	443.56 ± 14.60
HFD	434.56 ± 15.64 ^*∗*^	458.37 ± 24.34 ^*∗*^	465.61 ± 21.49 ^*∗*^	488.55 ± 19.39 ^*∗*^	504.94 ± 33.74 ^*∗*^	513.11 ± 30.71 ^*∗*^
Orlistat	433.67 ± 14.48#	417.4 ± 26.12#	411.89 ± 21.62#	400.13 ± 22.35#	392.79 ± 17.97#	381.78 ± 14.83#
FC-L	433.89 ± 16.71#	435.62 ± 21.61	444.19 ± 17.28#	452.32 ± 31.74#	448.24 ± 11.85#	449.33 ± 12.79#
FC-M	433.33 ± 14.61#	431.93 ± 16.92#	427.84 ± 27.18#	417.91 ± 30.69#	415.26 ± 20.04#	414.22 ± 25.04#
FC-H	433.67 ± 17.1#	428.45 ± 18.19#	419.7 ± 33.77#	412.67 ± 21.21#	398.92 ± 16.95#	396.44 ± 26.39#

Data are expressed as mean ± SEM (*n* = 10);  ^*∗*^*P* < 0.05 vs ND group; #*P* < 0.05 vs HFD group.

**Table 3:** Effects of FC on waist circumference each week during gavage.

	1th	2nd	3rd	4th	5th	6th
ND	14.91 ± 0.26	15.20 ± 0.23	15.31 ± 0.54	15.49 ± 0.40	15.74 ± 0.45	15.78 ± 0.42
HFD	16.36 ± 0.47 ^*∗*^	16.62 ± 0.47 ^*∗*^	16.68 ± 0.41 ^*∗*^	16.39 ± 0.57 ^*∗*^	16.40 ± 0.68 ^*∗*^	16.36 ± 0.58
Orlistat	16.21 ± 0.54	15.71 ± 0.49	15.46 ± 0.48#	15.34 ± 0.36#	15.04 ± 0.36#	14.77 ± 0.39#
FC-L	16.38 ± 0.45	16.33 ± 0.39	16.04 ± 0.33	15.77 ± 0.43	15.75 ± 0.42	15.73 ± 0.39#
FC-M	16.34 ± 0.31	16.25 ± 0.33	15.92 ± 0.26	15.85 ± 0.49	15.68 ± 0.40	15.51 ± 0.44#
FC-H	16.32 ± 0.37	16.10 ± 0.33	15.69 ± 0.41	15.61 ± 0.38	15.27 ± 0.39#	15.24 ± 0.32#

Data are expressed as mean ± SEM (*n* = 10);  ^*∗*^*P* < 0.05 vs ND group;  ^#^*P* < 0.05 vs HFD group.

## Data Availability

The datasets used and/or analyzed during the current study are available from the corresponding author on reasonable request.

## Abbreviations

FC: Finger citron
TGR5: G protein-coupled receptor 5
RT-PCR: Reverse transcription-polymerase chain reaction analysis
GLP-1: Glucagon-like peptide-1
ALT: Alanine aminotransferase
AST: Aspartate aminotransferase
TNF-*α*: Tumor necrosis factor-*α*
IL-6: Interleukin-6
NF-*κ*B: Nuclear factor-kappa B inhibitor kinase
SOD: Superoxide dismutase
MDA: Malondialdehyde
cAMP: Cyclic adenosine monophosphate
HepG2: Hepatocellular carcinoma cells
FBG: Fasting blood glucose
HDL-C: High-density lipoprotein cholesterol
LDL-C: Low-density lipoprotein cholesterol
MDA: Malondialdehyde
TG: Total triglycerides
TC: Total cholesterol
HE: Hematoxylin and eosin
ND: Normal diet group
HFD: High-fat diet group
FC-L: Low dose of FC
FC-M: Medium dose of FC
FC-H: High dose of FC
SD: Standard deviation.
